# Draft Genome Sequence of *Paenibacillus polymyxa* EBL06, a Plant Growth-Promoting Bacterium Isolated from Wheat Phyllosphere

**DOI:** 10.1128/genomeA.00414-15

**Published:** 2015-05-07

**Authors:** Shengxian Liang, Decai Jin, Xinxin Wang, Haiyan Fan, Zhihui Bai

**Affiliations:** a College of Bioscience and Biotechnology, Shenyang Agricultural University, Shenyang, China; b Research Center for Eco-Environmental Sciences, Key Laboratory of Environmental Biotechnology, Chinese Academy of Sciences, Beijing, China; c China Offshore Environmental Service Co. Ltd., Tianjin, China

## Abstract

*Paenibacillus polymyxa* strain EBL06 is a plant growth-promoting bacterium with high antifungal activity. The estimated genome of this strain is 5.68 Mb in size and harbors 4,792 coding sequences (CDSs).

## GENOME ANNOUNCEMENT

*Paenibacillus polymyxa* (formally *Bacillus polymyxa*) is a Gram-positive, endospore-forming bacterium belonging to a diverse group of plant growth-promoting bacteria (PGPB). Our previous study showed that *P. polymyxa* strain EBL06, isolated from wheat phyllosphere, had a high level of antagonistic fungus activity (1) and could stimulate tea plant growth and improve the quality of *Brassica rapa* L. *chinensis* (2, 3).

Here, we report the draft genome sequence of *P. polymyxa* EBL06. Genome DNA was extracted and sequenced using the Illumina HiSeq 2000 platform. Shotgun sequencing produced 17,775,760 paired-end reads with approximately 264-fold coverage. Filtered reads were assembled filled by SOAP*denovo* 2.04 (4). This assembly generated 102 contigs, with an *N*_50_ length of 627,482 bp and an average length of 55,594 bp, which were assembled into 45 scaffolds, with an *N*_50_ length of 1,481,754 bp and an average length of 126,218 bp. Genome annotation was performed by the NCBI Prokaryotic Genome Annotation Pipeline (PGAP) (http://www.ncbi.nlm.nih.gov/genome/annotation_prok/).

The draft genome is 5.68 Mb, with a G+C% content of 45.59%. A total of 4,792 coding sequences (CDSs), 105 pseudogenes, 41 tRNA genes, 1 noncoding RNA (ncRNA), and 9 rRNAs were identified.

Average nucleotide identity (ANI) analysis (5) revealed that *P. polymyxa* EBL06 is phylogenetically related to *P. polymyxa* SC2 (94.5%) (6), *P. polymyxa* M1 (94.5%) (7), *P. polymyxa* E681 (89.4%) (8), *P. polymyxa* SQR-21 (98.6%) (9), *P. polymyxa* CR1 (89.6%) (10), and *P. polymyxa* Sb3-1 (94.7%) (11).

### Nucleotide sequence accession number.

The whole-genome shotgun project of *P. polymyxa* EBL06 has been deposited at DDBI/EMBL/GenBank under the accession no. JYCW00000000. The version described in this paper is the first version.

## References

[1] Gu L, Bai Z, Jin B, Zhang J, Li W, Zhuang G, Zhang H 2010 Production of a newly isolated *Paenibacillus polymyxa* biocontrol agent using monosodium glutamate wastewater and potato wastewater. J Environ Sci China 22:1407–1412. doi:10.1016/S1001-0742(09)60267-9. 21174972

[2] Xu S, Bai Z, Jin B, Xiao R, Zhuang G 2014 Bioconversion of wastewater from sweet potato starch production to *Paenibacillus polymyxa* biofertilizer for tea plants. Sci Rep 4:4131. doi:10.1038/srep04131. 24576979 PMC3937799

[3] Su Y, Bai Z, Lv X, Zhang L, Peng X 2011 Effect of *Paenibacillus polymyxa* on nitrate content in rape. Chin Agric Sci Bull 27:144–148. (In Chinese.)

[4] Luo R, Liu B, Xie Y, Li Z, Huang W, Yuan J, He G, Chen Y, Pan Q, Liu Y, Tang J, Wu G, Zhang H, Shi Y, Liu Y, Yu C, Wang B, Lu Y, Han C, Cheung DW, Yiu SM, Peng S, Xiaoqian Z, Liu G, Liao X, Li Y, Yang H, Wang J, Lam TW, Wang J 2012 SOAP*denovo*2: an empirically improved memory-efficient short-read *de novo* assembler. Gigascience 1:18. doi:10.1186/2047-217X-1-18. 23587118 PMC3626529

[5] Richter M, Rosselló-Móra R 2009 Shifting the genomic gold standard for the prokaryotic species definition. Proc Natl Acad Sci U S A 106:19126–19131 doi:10.1073/pnas.0906412106. 19855009 PMC2776425

[6] Ma M, Wang C, Ding Y, Li L, Shen D, Jiang X, Guan D, Cao F, Chen H, Feng R, Wang X, Ge Y, Yao L, Bing X, Yang X, Li J, Du B 2011 Complete genome sequence of *Paenibacillus polymyxa* SC2, a strain of plant growth-promoting rhizobacterium with broad-spectrum antimicrobial activity. J Bacteriol 193:311–312. doi:10.1128/JB.01234-10. 21037012 PMC3019932

[7] Niu B, Rueckert C, Blom J, Wang Q, Borriss R 2011 The genome of the plant growth-promoting rhizobacterium *Paenibacillus polymyxa* M-1 contains nine sites dedicated to nonribosomal synthesis of lipopeptides and polyketides. J Bacteriol 193:5862–5863. doi:10.1128/JB.05806-11. 21952539 PMC3187208

[8] Kim JF, Jeong H, Park SY, Kim SB, Park YK, Choi SK, Ryu CM, Hur CG, Ghim SY, Oh TK, Kim JJ, Park CS, Park SH 2010 Genome sequence of the polymyxin-producing plant-probiotic rhizobacterium *Paenibacillus polymyxa* E681. J Bacteriol 192:6103–6104. doi:10.1128/JB.00983-10. 20851896 PMC2976442

[9] Li S, Yang D, Qiu M, Shao J, Guo R, Shen B, Yin X, Zhang R, Zhang N, Shen Q 2014 Complete genome sequence of *Paenibacillus polymyxa* SQR-21, a plant growth-promoting rhizobacterium with antifungal activity and rhizosphere colonization ability. Genome Announc 2(2):e00281-14. doi:10.1128/genomeA.00281-14. 24723719 PMC3983308

[10] Eastman AW, Weselowski B, Nathoo N, Yuan ZC 2014 Complete genome sequence of *Paenibacillus polymyxa* CR1, a plant growth-promoting bacterium isolated from the corn rhizosphere exhibiting potential for biocontrol, biomass degradation, and biofuel production. Genome Announc 2(1):e01218-13. doi:10.1128/genomeA.01218-13. 24459277 PMC3900909

[11] Rybakova D, Wetzlinger U, Müller H, Berg G 2015 Complete genome sequence of *Paenibacillus polymyxa* strain SB3-1, a soilborne bacterium with antagonistic activity toward plant pathogens. Genome Announc 3(2):e00052-15. doi:10.1128/genomeA.00052-15. 25767224 PMC4357746

